# Retrieving magma composition from TIR spectra: implications for terrestrial planets investigations

**DOI:** 10.1038/s41598-019-51543-9

**Published:** 2019-10-23

**Authors:** Alessandro Pisello, Francesco P. Vetere, Matteo Bisolfati, Alessandro Maturilli, Daniele Morgavi, Cristina Pauselli, Gianluca Iezzi, Michele Lustrino, Diego Perugini

**Affiliations:** 1 Dept. of Physics and Geology, University of Perugia, Piazza Università 1, 06123 Perugia, Italy; 2 Institute of Mineralogy, Leibniz Universität Hannover, Callinstr. 3, D-30167 Hannover, Germany; 3 Institute for Planetary Research, DLR, Rutherfordstrasse 2, 12489 Berlin-Adlershof, Germany; 4 Dipartimento di Ingegneria e Geologia, Università degli studi Chieti, Via dei Vestini – Campus Universitario, 66100 Chieti, Italy; 5 Dipartimento di Scienze della Terra, Università Roma La Sapienza, Piazzale Aldo Moro, 5, 00185 Roma, Italy; 6 Istituto di Geologia Ambientale e Geoingegneria (IGAG-CNR) c/o Dipartimento di Scienze della Terra, Università Roma La Sapienza, Piazzale Aldo Moro, 5, 00185 Roma, Italy; 7 INGV of Rome, Via di Vigna Murata, 605, 00143 Rome, Italy

**Keywords:** Petrology, Petrology

## Abstract

Emissivity and reflectance spectra have been investigated on two series of silicate glasses, having compositions belonging to alkaline and subalkaline series, covering the most common terrestrial igneous rocks. Glasses were synthesized starting from natural end-members outcropping at Vulcano Island (Aeolian Islands, Italy) and on Snake River Plain (USA). Results show that the shift of the spectra, by taking Christiansen feature (CF) as a reference point, is correlated with SiO_2_ content, the SCFM factor and/or the degree of polymerization state via the NBO/T and temperature. The more evolved is the composition, the more polymerized the structure, the shorter the wavelength at which CF is observable. CF shift is also dependent on temperature. The shape of the spectra discriminates alkaline character, and it is related to the evolution of Qn structural units. Vulcano alkaline series show larger amount of Q_4_ and Q_3_ species even for mafic samples compared to the subalkaline Snake River Plain series. Our results provide new and robust insights for the geochemical characterization of volcanic rocks by remote sensing, with the outlook to infer origin of magmas both on Earth as well as on terrestrial planets or rocky bodies, from emissivity and reflectance spectra.

## Introduction

A major challenge in planetary investigations is the remote determination of compositions of rocky planets’ surface, for the reason that this is a key feature to assess the geological evolution of that body^1^. As consequence of the difficulty to directly sample rocky planets and satellites (exception are Moon and, possibly, Mars), satellite data represent the only source of information. An important part of planetary studies is based on using spectroscopy to characterize analogue materials, in order to perform comparisons with spectroscopic data obtained with telescopic and/or *in situ* methods. On this concern, spectral response of geologically relevant samples has been investigated in various ranges: visible (VIS), near infrared (NIR)^2–4^, mid infrared (mid-IR) and thermal infrared (TIR)^5,6^, or for other vibrational spectroscopy techniques^7,8^. At present, the interpretation of spectroscopic data mainly involves models calibrated using single minerals^9,10^ or rocks^11,12^. These models are thus useful^3,13^ but incomplete in cases of amorphous materials, such as hypohyaline to obsidianaceous volcanic rocks. Consequently, existing models cannot be automatically extrapolated to formulate high-resolution hypotheses, especially when volcanic activities are frequent and widespread. In fact, effusive and explosive volcanism extensively occurred on rocky planets and satellites (Mercury, Moon, Mars, Io, Vesta), covering huge areas of their surfaces and shaping peculiar morphology^14,15^. Therefore, the study of the chemical composition of extraterrestrial volcanism is of great importance to infer the geological history of Solar System rocky bodies, only if non-crystalline fractions are correctly accounted.

Spectral research on silicate glasses was indeed performed in past times, with various aims. Glasses were spectroscopically investigated in the mid-IT and TIR for general material characterization^16–21^, but also for purposes related to planetary investigations: glasses were used to characterize basaltic compositions on Mars and on the Moon^22,23^, to characterize alteration of tephra on planetary surfaces^24,25^, to investigate possible silica coatings on planetary terrains^26–28^, or to focus on spectral feature of possible impact glass^29^. However, no study exists focusing on glass as a proxy for magmatic bodies on planetary surfaces, especially for what concerns variation of spectral response of silicate glasses with evolving compositions (from mafic to silicic) or between different magmatic series (from subalkaline to alkaline).

In this study, we propose an interpretation of spectra regarding two sets of glass compositions, representing different magmatic series, showing variability in SiO_2_ content (which defines compositional evolution) and in alkali content (which on Earth can be associated to specific geodynamic settings). We focus on the TIR region, in particular for what concerns the wavelength range from 7 to 14 *µ*m, with the aim to identify possible systematic variations that might be used to in depth interpretation of spectroscopic data.

In 2018 the BepiColombo mission was launched to Mercury, carrying the Mercury Radiometer and Thermal Infrared Spectrometer (hereon, MERTIS), whose goal is to map the entire planet with ~500 m spatial resolution and ~90 nm spectral resolution for a wavelength range from 7 to 14 *µ*m^30^. Mercury surface is thought to be widely covered by products from effusive volcanism in its northern hemisphere, known as Northern Smooth Plains (NSP)^31^ or Northern Volcanic Province (NVP)^32^, but additional volcanism-related features are present on other regions^33,34^. Thus, Mercury could represent a challenging example for the interpretation of TIR spectroscopic data from volcanic terrains, related to both effusive and explosive volcanism.

## Results

**Figure 1:**
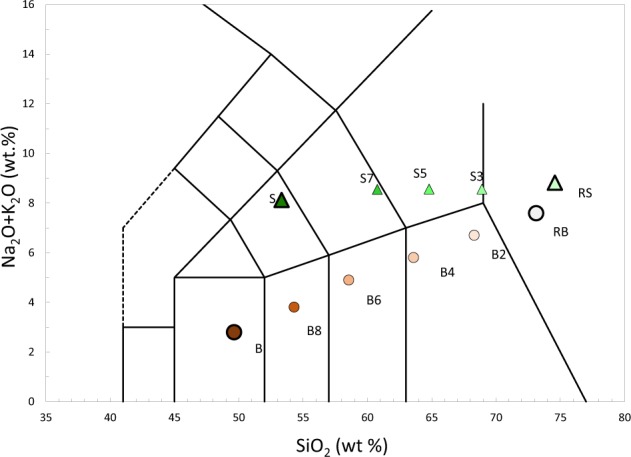
Total alkali-silica diagram (TAS)^61^ for end-members and intermediate compositions used in this study. Compositions are normalized on anhydrous basis. S and RS refer to the Vulcano Island shoshonite and rhyolite end-members, while B and RB refer to the Snake River Plain basalt and rhyolite end-members. Details are reported into the main text.

Two sets of glasses whose composition resembles the ones of two magmatic series were synthesized and spectrally characterized in the TIR range. Methodologies are extensively described in the Methods section at the end of this paper. The major oxide chemical composition of experimental samples is reported in Table 1. It is observable how every parameter is linearly varying from an end-member to the other. Thus, we can state that mixing of glasses was successful, resulting in the expected regular variation of chemical compositions as shown in Fig. 1. Calculated iron speciation is reported on Table 2.

**Table 1:** Chemical compositions of synthesized glasses reported as an average of multiple measurements for each sample together with Standard deviation (S.D.).

Sample (no of analyses)		SiO_2_	TiO_2_	Al_2_O_3_	FeO	MnO	MgO	CaO	Na_2_O	K_2_O	P_2_O_5_	Cl	Total
S	Wt. %	53.33	0.65	15.29	7.63	0.16	4.15	7.76	5.08	3.04	0.50		97.61
(10)	S. D.	0.34	0.02	0.12	0.17	0.03	0.08	0.14	0.11	0.06	0.04		
S7	Wt. %	59.66	0.48	14.55	5.94	0.13	2.85	5.66	4.63	3.77	0.33	0.17	98.18
(8)	S. D.	1.03	0.05	0.30	0.08	0.02	0.05	0.05	0.10	0.06	0.03	0.02	
S5	Wt. %	64.08	0.38	14.03	4.86	0.12	2.11	4.38	4.32	4.15	0.29	0.16	98.90
(8)	S. D.	0.46	0.02	0.22	0.10	0.03	0.05	0.10	0.06	0.05	0.03	0.03	
S3	Wt. %	67.96	0.28	13.40	3.73	0.08	1.37	3.05	3.96	4.49	0.17	0.16	98.63
(8)	S. D.	0.65	0.02	0.30	0.10	0.03	0.04	0.08	0.04	0.07	0.04	0.02	
RS	Wt. %	73.96	0.11	12.79	1.98	0.05	0.21	1.07	3.63	5.12	0.04	0.22	99.18
(8)	S. D.	0.70	0.02	0.14	0.05	0.02	0.02	0.05	0.08	0.10	0.03	0.03	
B	Wt. %	48.03	1.68	15.01	10.98	0.17	7.30	10.55	2.39	0.32	0.35	0.02	96.79
(4)	S. D.	0.24	0.03	0.29	0.08	0.04	0.12	0.07	0.09	0.02	0.03	0.01	
B8	Wt. %	53.03	1.48	14.63	9.67	0.16	5.93	8.74	2.52	1.20	0.29	0.01	97.66
(7)	S. D.	0.34	0.04	0.29	0.20	0.03	0.09	0.08	0.08	0.03	0.02	0.01	
B6	Wt. %	57.07	1.30	14.09	8.36	0.12	4.50	7.00	2.69	2.09	0.25	0.01	97.47
(7)	S. D.	0.45	0.04	0.25	0.16	0.03	0.07	0.05	0.05	0.05	0.04	0.01	
B4	Wt. %	62.64	1.06	13.53	6.89	0.10	3.17	5.20	2.74	2.98	0.21	0.01	98.52
(7)	S. D.	0.41	0.04	0.22	0.10	0.04	0.07	0.10	0.09	0.05	0.02	0.01	
B2	Wt. %	67.47	0.82	13.03	5.42	0.07	1.70	3.44	2.79	3.83	0.20	0.01	98.79
(7)	S. D.	0.66	0.04	0.20	0.11	0.01	0.06	0.06	0.06	0.05	0.03	0.01	
RB	Wt. %	72.28	0.63	12.53	3.87	0.03	0.25	1.61	2.77	4.74	0.16	0.00	98.86
(7)	S. D.	0.65	0.05	0.24	0.15	0.02	0.01	0.03	0.06	0.06	0.03	0.00	

In brackets the number of analyses for each sample.

**Table 2:** Various parameters calculated for each sample.

	NBO/T	FeO/Fe_tot_	FeO (wt.%)	Fe_2_O_3_ (wt.%)	SCFM
S	0.36	0.35	2.74	5.08	0.79
S7	0.24	0.34	2.06	3.99	0.85
S5	0.17	0.33	1.64	3.28	0.89
S3	0.11	0.33	1.23	2.55	0.92
RS	0.02	0.32	0.63	1.37	0.97
B	0.50	0.36	4.07	7.27	0.69
B8	0.39	0.36	3.54	6.36	0.75
B6	0.29	0.35	3.03	5.55	0.80
B4	0.20	0.35	2.41	4.58	0.85
B2	0.11	0.33	1.83	3.66	0.91
RB	0.02	0.30	1.17	2.74	0.93

NBO/T is calculated with Eq. 1, Fe speciation is calculated taking into consideration air fugacity^59^,SCFM is calculated with Eq. 2.

### Spectroscopic characterization: reflectance and emissivity

**Figure 2:**
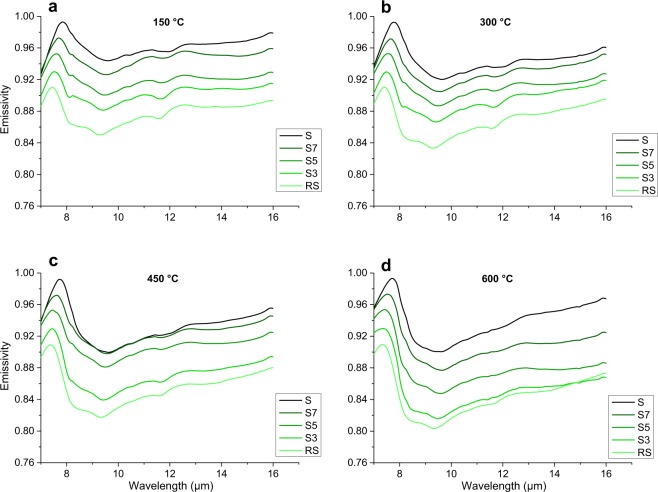
Emissivity spectra collected for the Vulcano (S-RS) series in the 7–16 µm wavelength range. Each sub-figure represents the emissivity of the entire set of glasses, collected at (**a**) 150 °C, (**b**) 300 °C, (**c**) 450 °C, and (**d**) 600 °C. Spectra are stacked in order to better highlight differences, with a vertical offset of −0.02, starting from the mafic endmember (darkest, at the top) down to the most evolved composition (lightest, at the bottom).

**Figure 3:**
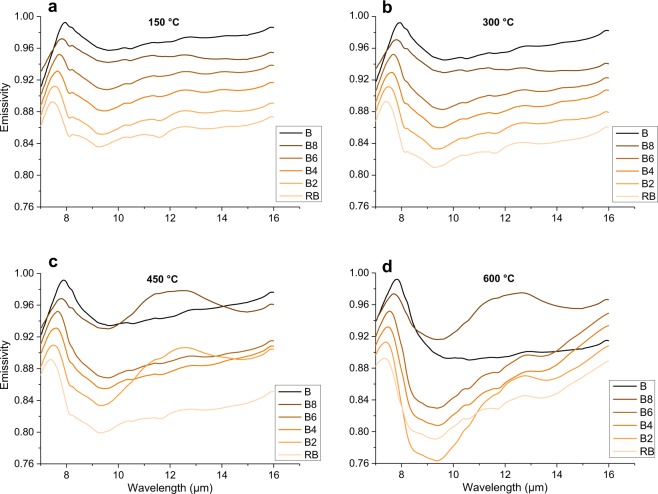
Emissivity spectra collected for the Snake River Plain (B-RB) series in the 7–16 µm wavelength range. Each sub-figure represents the emissivity of the entire set of glasses, collected at (**a**) 150 °C, (**b**) 300 °C, (**c**) 450 °C, and (**d**) 600 °C. Spectra are stacked in order to better highlight differences, with a vertical offset of −0.02, starting from the mafic endmember (darkest, at the top) down to the most evolved composition (lightest, at the bottom).

**Figure 4:**
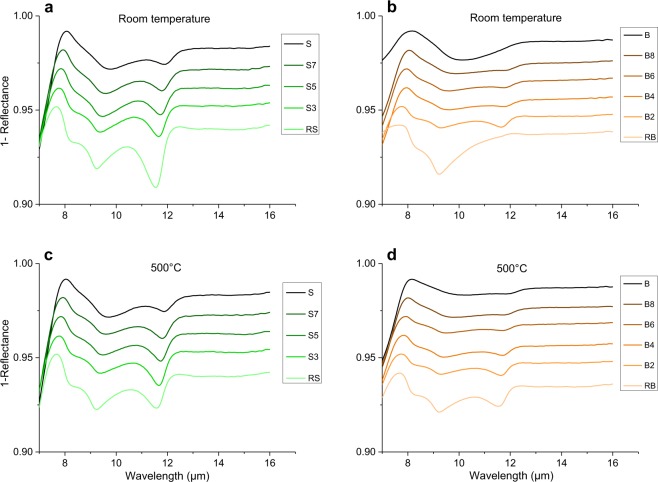
Reflectance spectra collected for (**a,c**) Vulcano (green spectra), and (**b,d**) Snake River Plane (orange spectra) series. Data are collected in 7–16 µm wavelength range at (**a,b**) room temperature (R.T.) and (**c,d**) 500 °C. Spectra are stacked in order to better highlight differences, with a vertical offset of −0.01, starting from the mafic endmember (darkest, at the top) down to the most evolved composition (lightest, at the bottom).

Figures 2 and 3 show the acquired emissivity spectra for the Vulcano and the Snake River Plain glasses, respectively, for different acquisition temperature. This study will focus on the spectral range between 7 and 14 *µ*m wavelength. In Fig. 4, reflectance spectra are reported, acquired at 20 °C (room temperature, hereon R.T.) and 500 °C. Spectra are shown as 1-reflectance (1-R), for a better comparison with emissivity.

All the spectra present a peak of maximum emissivity (minimum reflectance) at ~8 *µ*m, where emissivity is close to the unity and reflectance is close to zero. This spectral feature is related to Christiansen effect, which appears at the wavelength at which the material refractive index approaches the refractive index of the medium, resulting in a backscattering minimum^35^. We are able to refer at this emissivity maximum caused by Christiansen effect as the Christiansen Feature (hereon CF, also present in literature as Christiansen maximum)^3,36^. Other works focusing on emissivity and or reflectance of silicates have individuated CF as a useful spectral feature^37–39^, and it is particularly efficient when glasses are taken into account, because of the scarcity of spectral features in amorphous materials^10,40,41^.

The range between the CF and ~14 *µ*m wavelength is the area where main *reststrahlen* bands, linked to vibrations of bonds regarding 4-coordinated oxides (such as Si and Al), appear^16,19,20^. Beyond CF, almost all our spectra, with the exception of sample B, present an evident feature between 11.5 and 12 *µ*m as an emissivity minimum/reflectance maximum. This is a diagnostic feature, the Transparency Feature, (hereon, TF)^6^ which is characteristic for extremely fine samples^42^. Appearance of TF is linked to the surface/volume ratio of a particle, which defines the ratio of the amount of scattered/absorbed light^43^. This feature is observed to be stronger for felsic minerals^44^ and we observe TF to be stronger for more evolved compositions even for our dataset of glasses. Even if TF may overprint our spectral information, it is of great interest as such a feature is possible to be observed in extraterrestrial samples, and it might be a useful feature to detect fine-grained terrains and relative evolution of chemical composition.

Thus, two are the main spectral characteristics on which we will focus: the shift in wavelength of spectra by taking CF as a reference point, and the change in the features that determine the shape of the spectra in the 7–14 *µ*m region.

#### Shift of the spectra

For what concerns reflectance, a gradual shift of the entire spectrum from longer to shorter wavelengths is observed with increasing SiO_2_ content (Fig. 4). By using CF as a reference point, this shift can be quantitatively determined. CF shows shifts of ~0.5 *µ*m throughout the entire series. The shift appears to be slightly shorter for the Vulcano series, which indeed has a slightly more restricted SiO_2_ range (Table 1 and Fig. 1).

For emissivity spectra, CF composition-dependent shift consists of ~0.5 *µ*m for each series. Moreover, a temperature-dependent shift is observed, it appears to be weaker than compositional-driven shift.

#### Shape of the spectra: from CF to 14 µm

By taking into consideration reflectance spectra, Snake River Plain series shows weaker intensity of reflectance and smoother shape of the spectra (Fig. 4b). B sample spectrum shows a unique protuberance rather than a peak, roughly centered at ~10.2 *µ*m. A substantial spectral shape change is observed as chemical composition varies towards more evolved, SiO_2_-richer, compositions. B8 composition shows two peaks maxima at ~9.8 and ~11.8 *µ*m (TF), with a smaller shoulder at ~8.5 *µ*m. As we move again towards SiO_2_-richer compositions these peak features become clearer and more dominant, and a shift towards shorter wavelengths is observed. The rhyolitic end-member (RB) shows a large peak centered at ~9.3 *µ*m with two small shoulders, respectively at ~8.3 *µ*m and TF at ~11.7 *µ*m.

For what concerns reflectance of Vulcano series (Fig. 4a), two peaks at ~9.7 *µ*m and TF at ~11.9 *µ*m and a small shoulder at ~8.7 *µ*m are observed for the mafic end-member S. As we move towards more evolved SiO_2_-richer compositions, these features shift towards shorter wavelengths and become gradually more evident and distinguishable, until clearly showing three peaks at ~8.2 *µ*m, ~9.3 *µ*m and TF at ~11.5 *µ*m for the more silicic rhyolitic end-member.

For both series of reflectance spectra, the increase in temperature of ~500 °C (Fig. 4c,d) has not a strong influence on the shape of the spectra for Vulcano series, whereas for Snake River Plain series an identical increase in temperature results in a stronger contrast of the spectra. In particular, three peaks are much more clearly distinguishable for the two end-members B and RB. Qualitatively, we observe a more evident compositionally-dependent change in the shape of the reflectance spectra for the Snake River Plain relative to Vulcano samples. These latter show, instead, a gradual and slight transition.

For what concerns the sets of emissivity spectra, their shape overlaps when similar compositions and different temperature are taken into consideration (Figs 2 and 3). However, a general absorption increase is observed with no great effect on the shape of the spectra: this can be appreciated in particular when taking into account local emissivity minimum at ~9–9,5 *µ*m, that deepens with increasing temperature. Exceptions are B2 and B8 compositions, at 450 and 600 °C (Fig. 3). The general temperature-dependent increase in absorption does not seem to be linearly correlated with the increase of temperature: emissivity spectra obtained at 300 and 450 °C are very similar, with a bold step when increasing up to 600 °C. By changing the chemical composition at constant temperature, the observations that can be made for the evolution of emissivity spectra are very similar to the ones that were made for the evolution of reflectance spectra. Peaks and features detected with reflectance are still present in the emissivity spectra, with identical trends, but TF is slowly disappearing with increasing temperature. Moreover, there are more features detectable for the emissivity spectra rather than reflectance spectra. As an example, close to 8.3–8.5 *µ*m, two features (instead of only one observable in the reflectance spectra) can be noticed: (i) a peak at slightly shorter wavelengths, and (ii) a bulge at slightly longer wavelengths (Figs 2–4). A minor peak can be noticed at 10.5 *µ*m for all emissivity spectra. It does not appear to show relevant variations as a function of temperature and/or chemical composition.

## Discussion

Electromagnetic waves interact with solid matter causing molecules to vibrate by changing the bond lengths and/or the angle between different bonds. This constitutes the basis for vibrational spectroscopy and its applications^10,16^, resulting in the fact that wavelengths of absorption/emission are determined by different bond lengths. The whole infrared region (IR) is the range where phenomena relative to the absorption/emission of energy due to vibration of bonds largely occur. The general model used to describe vibrational behavior of bonds is the anharmonic quantum oscillator^45^. This model establishes that bonds display a discrete number of vibrations when hit by electromagnetic wave. Therefore, there is a discrete number of wavelengths that are absorbed/emitted due to bond vibration. Crystals are long-range ordered materials easily investigable through diffraction methods, therefore they have furnished exhaustive interpreted databases. Conversely, amorphous/glassy materials are not investigable by diffraction methods, and detailed studies resulting in systematic reference data are consequentially scarce^9,30,35^.

Although glasses lack long-range order, short or medium range order are present as a structural feature in the overall chaotic geometrical arrangement of the elements, allowing us to use vibrational spectroscopy on glasses^46^. Interpretation of the IR spectra from glasses has been proposed in literature^40,41^ mainly concerning synthetic simple compositions related to earth as well as terrestrial planets^17–29^.

In a silicate glass, SiO_2_ tetrahedra are the dominant structural feature. These tetrahedra are linked by bonding oxygens with each other or with other molecular clusters with different geometrical arrangements. These short-range features define the most important physical properties of the system, such as viscosity and, consequently, chemical diffusivity^47–49^. Moreover, other elements (such as Al, Fe, Ti, etc.) may coordinate with 4 oxygens, under specific conditions. These elements are known as network former cations (hereafter, T), whereas other cations, which are not 4-coordinated with oxygen, are called network modifiers^50^. The behavior of alkali elements (Na and K) in relationship with Al and Fe^2+^/Fe_tot_ determines important structural features of glasses: alkalis may act as network balancers for Al and Fe, so that the tetrahedrical structure is not interrupted and T-O bonds become longer^15,16,51^. Since alkalis have such an important influence on the structure, we have chosen to investigate series with substantial difference in alkali content (Table 1).

In order to determine polymerization degree of the studied compositions, we use the NBO/T parameter defined as:

1 $$\frac{NBO}{T}=\frac{O-T}{T}$$

where NBO stands for non-bridging oxygen (the number of oxygen atoms not linking two network formers)^52^, O represents total oxygen moles and T represents total moles of all cations acting as network formers. As for Al and Fe^3+^, only moles exceeding that of alkaline (Na, K) and earth alkaline cations (Mg, Ca) are considered network formers and therefore included in the calculation for T^50^. Thus, this parameter takes into account not only the role of the classic network builders such as Si, but also other cations which are influenced by alkalis and earth alkalis.

Another useful parameter to be used for quantifying the structural properties of glasses is the so-called SCFM parameter (taking its name from the oxides involved in the definition: SiO_2_, CaO, FeO, MgO)^10^,and defined in mass proportion as:

2 $$SCFM=\frac{Si{O}_{2}}{Si{O}_{2}+CaO+FeO+MgO}$$

This parameter takes into account SiO_2_, which is the main network former, and the most important divalent cations, which are considered as the main network modifiers. Since its definition, SCFM parameter has been widely used to build up interpretative models for planetary science^11,36,42^. However, this is a mere empirical parameter linking chemical composition and spectral response, and has no structural meaning for amorphous material such as glasses. Calculated NBO/T and SCFM are reported on Table 2.

**Figure 5:**
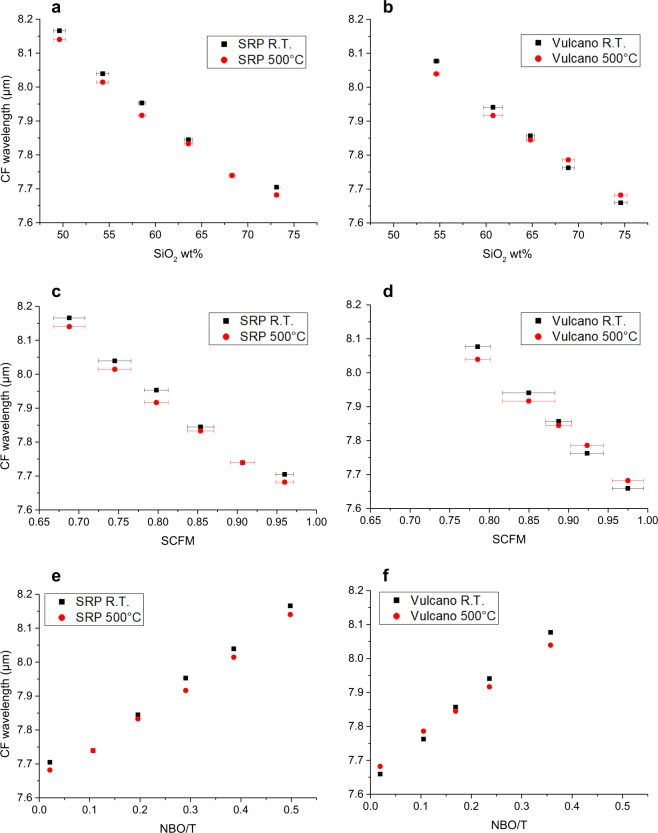
Particular for the Snake River Plain (SRP) and Vulcano series Christiansen Feature CF vs. (**a, b**) SiO_2_, (**b, c**) SCFM and (**c, d**) NBO/T related to reflectance spectra. All plots well correlated with a linear relationship whose R^2^ and slope are provided in Table 3. Please refer to main text for details and discussions.

**Figure 6:**
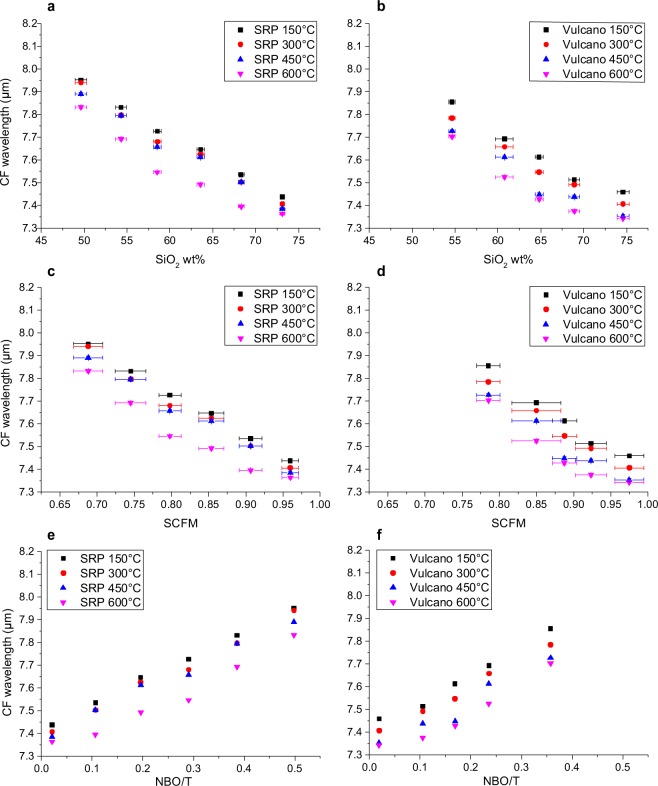
Particular for the Snake River Plain (SRP) and Vulcano series Christiansen Feature CF vs. (**a, b**) SiO_2_, (**b, c**) SCFM and (**c, d**) NBO/T related to emissivity spectra. All plots well correlated with a linear relationship whose R^2^ and slope are provided in Table 3. Please refer to main text for details and discussions.

The shift towards shorter wavelengths moving from the less evolved (mafic) to the more evolved (felsic) compositions is one of the most striking features observed in our set of spectra (Figs 2–4). This phenomenon is well known^16^, and has been interpreted as a potential marker for the evolution of the tetrahedral network within the structure, or the so-called “degree of polymerization”. From computer simulations and experimental studies^18,46^ we know that, as tetrahedra link to each other, the average T-O bond distance becomes shorter. Thus, we compare the shift in our spectra with: (1) SiO_2_ content, because it is the main network former, (2) SCFM, which is a widely used parameter correlating SiO_2_ to divalent cations, which are the main network modifiers, and (3) NBO/T parameter, which is a parameter quantifying the presence of network forming/network modifiers elements in the structure. Figures 5 and 6 show the relationship between shift of CF (for reflectance and emissivity, respectively) in relationship with SiO_2_ content, SCFM and NBO/T for each of the experimental sample.

If we focus on reflectance shifts reported in Fig. 5, the same linear relationship is observed for all the series. As the SiO_2_ increases, SCFM increases, NBO/T decreases, and CF shifts towards shorter wavelengths. If only SRP series is taken into account, we observe that the increase in temperature shifts CF to shorter wavelengths, without modifying the slope of the general linearity. This is true for all the considered experimental SRP data. On the contrary, the Vulcano series present a slightly different slope for the two series acquired at room temperature and at 500 °C: the two trends intersect at ca. SiO_2_ = 65 wt%, SCFM = 0.89 and NBO/T = 0.18 (Fig. 5 b,d,f).

For SRP series, whose chemical composition strongly varies along the series in terms of SiO_2_ and alkali content, the increase in temperature seems to involve equally all compositions in the shift of CF. For Vulcano series, whose chemical composition does not strongly vary in alkali content, (Table 1 and Fig. 1) temperature is differently influencing the shift in CF for mafic and felsic compositions. Indeed, considering the SiO_2_-richest glasses of the Vulcano series, an increase in temperature produces a shift of the spectra towards longer wavelengths.

If we focus on emissivity shifts reported in Fig. 6, the shift trends are identical to those observed for reflectance, but generally shifted to slightly shorter wavelengths. An increase in temperature is always shifting the spectra towards shorter wavelengths. This effect is slightly larger for intermediate compositions and for the 450–600 °C step, resulting in a curvature of the series for higher temperatures. Table 3 reports correlation coefficients R^2^ and linear slopes for the data shown in Fig. 6. For emissivity, R^2^ slightly decreases as temperature increases, together with a light decrease in the slope of the trends. Differently to reflectance, the change in the slope of the series is not deeply different for the two series.

**Table 3:** Equations obtained from linear interpolation of scattered datasets together with their relative coefficients of determination R.

		SRP SiO_2_	Vulcano SiO_2_	SRP SCFM	Vulcano SCFM	SRP NBO/T	Vulcano NBO/T
Reflectance R.T.	Eq.	CF = −0.0201x + 9.14	CF = −0.0211x + 9.2245	CF = −1.7463x + 9.3489	CF = −2.23x + 9.8309	CF = 1.0016x + 7.6578	CF = 1.2493x + 7.6378
	R²	0.9813	0.9984	0.9855	0.9986	0.9904	0.9977
Reflectance 500 °C	Eq.	CF = −0.0194x + 9.077	CF = −0.0177x + 8.997	CF = −1.6863x + 9.2791	CF = −1.8693x + 9.5065	CF = 0.9675x + 7.6461	CF = 1.0473x + 7.668
	R²	0.9853	0.9972	0.9903	0.9994	0.9957	0.9986
Reflectance (both temperature)	Eq.	CF = −0.0198x + 9.1085	CF = −0.0194x + 9.1107	CF = −1.7163x + 9.314	CF = −2.0496x + 9.6687	CF = 0.9846x + 7.652	CF = 1.1483x + 7.6529
	R²	0.979	0.9897	0.9836	0.9908	0.9887	0.99
Emissivity 150 °C	Eq.	CF = −0.0215x + 9.0037	CF = −0.0202x + 8.9344	CF = −1.861x + 9.2247	CF = −2.1442x + 9.5232	CF = 1.065x + 7.4232	CF = 1.2052x + 7.4138
	R²	0.996	0.9712	0.9979	0.9799	0.9982	0.9855
Emissivity 300 °C	Eq.	CF = −0.0219x + 8.9976	CF = −0.0192x + 8.8206	CF = −1.8969x + 9.2243	CF = −2.035x + 9.3774	CF = 1.087x + 7.3876	CF = 1.1419x + 7.3757
	R²	0.9867	0.9837	0.9899	0.9892	0.9929	0.9915
Emissivity 450 °C	Eq.	CF = −0.021x + 8.9255	CF = −0.0191x + 8.7545	CF = −1.8174x + 9.1415	CF = −2.031x + 9.3125	CF = 1.0394x + 7.3823	CF = 1.1408x + 7.3145
	R²	0.9867	0.9365	0.9887	0.9454	0.9877	0.9494
Emissivity 600 °C	Eq.	CF = −0.0199x + 8.7702	CF = −0.0182x + 8.6533	CF = −1.7289x + 8.981	CF = −1.9442x + 9.1944	CF = 0.9954x + 7.3059	CF = 1.0967x + 7.281
	R²	0.9466	0.9112	0.957	0.9307	0.9689	0.9427

Here, CF is shown as dependent on *x*, which is respectively SiO2 wt%, SCFM or NBO/T.

For emissivity spectra, interpolation was performed only by taking into account each acquisition temperature separately. For reflectance, interpolation was performed by taking into account two different acquisition temperatures both as two different datasets (first two rows in the table) and as a unique dataset (third row).

The shape of the spectra between the CF and ~14 *µ*m relates on the vibration of T-O bonds, in particular Si-O and Al- O^16^. Reflectance spectra presented in Fig. 5 clearly show the difference between the two analyzed series, especially for room temperature (R.T.) acquisitions. For tetrahedral Si, the larger the amount of BO, the shorter the average Si-O distance, and the shorter the absorption/emission wavelength. Therefore, it appears reasonable that the most evolved chemical compositions will show features relative to shorter wavelengths. There are reviews^16^ reporting the approximate wavelengths where, despite the observed shift, vibrations of T-O bonds occur in relationship with the type of structural unit Qn^52^. For Q_4_ two vibrational regions are reported around 8–8.3 *µ*m and 9–10 *µ*m, Q_3_ shows vibration at 9–10 *µ*m, Q_2_ at 10–11 *µ*m, Q_1_ and Q_0_ at 11–12 *µ*m. The fact that in all the reflectance spectra the shoulder around 8.4 *µ*m is increasing with SiO_2_ content allows us to assign this feature to Si-bearing Q_4_ species. The progressive increase of the peak at ~9.5 *µ*m can also be interpreted as a consequence of the increase of Q_4_ and Q_3_ species. Moreover, the concave shape visible between 10 *µ*m and TF can be related to a decrease of Qn. Identical observations can be made for emissivity spectra at ~8.3–8.5 *µ*m. As reported above, the feature showing shorter wavelength is increasing as the chemical composition becomes richer in SiO_2_.

The development of spectral features when moving from mafic to felsic compositions, is clearly more marked for the Snake River Plain than for the Vulcano series, whose spectra are much more similar to each other. If we look at the TAS diagram in Fig. 1 it is clear that the SiO_2_ range is roughly overlapping for the two series. By observing chemical composition variations (Table 1) we observe how Al_2_O_3_ and the total Fe vary coherently between the two series. Therefore, the difference between the two sets of spectra may not be explained by taking into consideration variations of Fe, Al or Si oxides only. In fact, detailed analyses of the chemical composition of the two series indicate that the only substantial compositional difference is related to the alkali content. Indeed, the Vulcano series shows a roughly constant alkali content between 8 and 9 wt%, whereas Snake River Plain samples show an increase in alkalis with SiO_2_. In particular, for the Vulcano series, the decrease in Na_2_O from 5.08 to 3.63 wt% is compensated by an increase in K_2_O from 3.04 to 5.12 wt%, whereas for Snake River Plain series the Na_2_O content is slightly increasing from 2.39 to 2.77 wt% and K_2_O shows a strong increase from 0.32 up to 4.74 wt%. Alkali-O bonds do not show vibration in the investigated range, but, as mentioned above, they play an important role in determining coordination of cations determining their role as network former or modifier, and therefore influencing Qn speciation. Therefore, a fundamental difference between the two series concerning how much are secondary network formers like Fe and Al contributing to polymerization emerges. This might explain why spectral shapes from the two series appear different in spite of their SiO_2_ ranges looking similar.

The increase in temperature of experimental samples shows the typical effects of decrease in contrast and band shifting already observed in literature^52^. Previous studies have reported how the efficiency of this effect depends on features such as thermal conductivity and granulometry^30,53^.

By focusing on reflectance, temperature-driven shift towards shorter wavelengths appears for all chemical composition of the Snake River Plain series with the exception of B2. On the other hand, for Vulcano series, the felsic compositions show that an increase in temperature is shifting the spectra towards longer wavelengths. A similar phenomenon is observable for SRP series for B2 but not for RB (Figs 5 and 6). It is not clear whether this should be taken into account as an artifact or if it might be correlated to some particular low energy chemical reaction involving charge-balancing cations related to alkaline content.

**Figure 7:**
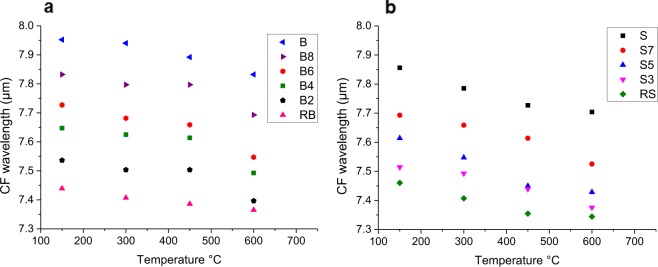
Position of CF for emissivity spectra acquired at different temperature for (**a**) SRP and (**b**) Vulcano. Composition-driven shift can be appreciated vertically.

As for emissivity spectra, thermal increase is definitely more efficient in determining a CF shift towards shorter wavelengths. In Fig. 7, the temperature-driven CF shift can be appreciated for all the emissivity spectra. It is clear that, even if not negligible, temperature-driven shift is decidedly weaker than composition-driven shift.

Figures 5–7 also show how temperature-driven CF shift is larger for the intermediate compositions, especially in the range 450–600 °C. This generates a slight non-linearity of the trends (Fig. 5), particularly evident for the Vulcano series. This non-linearity can be correlated to experimental routine bias: even if we are able to see at what temperature we are acquiring the spectra, we do not know the exact temperature of the emitting body itself. Moreover, the extent of temperature-driven spectral shift is slightly larger for glasses with higher alkali content.

From the spectra observable in Figs 4–6, we can calculate linear interpolation equations describing the CF shift with the three parameters taken into account. In Table 3, results from linear interpolation of scattered datasets are shown, taking into consideration (i) reflectance at both different acquisition temperature, (ii) reflectance without taking into account different acquisition temperature and (iii) emissivity at each different acquisition temperature. In this way, it is possible to use these equations to recalculate SiO_2_ content, SCFM and NBO/T. In Supplementary Tables (S1–S4), recalculated parameters are reported. For what concerns reflectance (Supplementary Table S1), the majority of recalculated SiO_2_ wt% content present a discrepancy which is lower than 1 wt% with the EPMA results, with few outliers for SRP trends whose discrepancy is however lower than 2 wt% (RB, B2, B and B4). This results in recalculated SCFM and NBO/T to present a discrepancy which is within the order of the second decimal for both parameters.

As already evidenced, reflectance scatters were also interpolated without taking into account different acquisition temperature. This was done because no large discrepancy of CF shifts when reflectance is acquired at room temperature or at 500 °C has been observed. By performing recalculation of parameters, discrepancies between EPMA and recalculated SiO_2_ content are lower than 2%, with only two exceptions (B and RB) however lower than 3% (Supplementary Table S2).

For what concerns emissivity spectra, recalculation of parameters is still very efficient for low temperature acquisitions, getting less precise when increasing temperature. However, for emissivity measured at 600 °C, discrepancy between calculated and measured SiO_2_ content is still always lower than 3% over a total SiO_2_ span of ~20% (Supplementary Tables S3 and S4).

Recalculations are far to be as precise as laboratory data, but they demonstrate that it is possible and relatively easy to distinguish between possible glass-bearing magmatic outcrops on other rocky planets, only by looking at the CF shift. As stated, Si content, SCFM and NBO/T are parameters that tell us about petrology of a planet and might help the community to shed light on the origin of magma on other planets.

## Conclusions

A first level characterization shows that shifting of the emissivity and reflectance spectra are readily parameterized by the silica content (SiO_2_ wt.%), the SCFM factor and the degree of polymerization NBO/T. In particular, Christiansen Feature (CF) is chosen as a characteristic signal correlated with these parameters: the more evolved the composition, the more polymerized is the structure, and the shorter is the wavelength at which the CF is observable. CF shift is not strongly influenced by alkali content or other chemical features besides SiO_2_, but it can be influenced by temperature.

A model to calculate SiO_2_ content, SCFM and NBO/T is proposed for both reflectance and emissivity, at different temperatures, resulting in a possibility to determine SiO_2_ content within an error of ~2 wt.% by only knowing CF wavelength.

The shape of the spectra between the CF and ~14 *µ*m is related to the evolution of structural units Qn which is different for the two series taken into consideration. Vulcano alkali series shows a large amount of Q_4_ and Q_3_ species, even for the most mafic samples. Difference in alkali content results in the spectroscopic response to be different making the two series clearly distinguishable. Being able to assess the alkaline character of magmatic bodies is of primary importance to understand volcanic features and to infer possible origin of magmas.

The wavelength range on which this study focuses is the same covered by MERTIS thermal infrared imaging spectrometer, mounted on the Mercury Planetary Orbiter for the BepiColombo Mission that is planned to first flyby Mercury in 2021, and finally reach its target late 2025 for two years of nominal mission. Mercury Northern Volcanic Province represents a huge example of extraterrestrial volcanic province. Here differences in granulometry and/or presence of crystals might also influence the interpretation of the acquired spectra. The long-term outlook of the present study is to start an interpretation of spatial remote data, which would not be based on the bare comparison of spectra, but rather on exhaustive databases where the features are interpreted from a physical and chemical point of view. Our idea is that in a near future, with the creation of an exhaustive database for planetary systems, an interpretation and decoding of spectroscopic data will be possible, to directly link them to specific chemical compositions. This would not only involve the study of amorphous rocks but also amorphous material coexisting with crystalline and/or vesicles formed under relevant geological conditions. As such, these results might be considered as a starting point towards the interpretation of evolutionary paths of magmas from other extraterrestrial rocky bodies. Further studies on chemical composition dependence related to the thermal shift should be performed in order to build up a solid database capable of being used for planetary investigation in future mission and to create a general model able to retrieve chemical composition from spectral data.

## Methods

### Sample preparation

Materials were synthesized starting from four natural rocks (end-members) collected at the Island of Vulcano (Aeolian Islands, Italy) and the Snake River Plain (USA). The first two end-members are shoshonite and rhyolite emplaced above an active subduction zone in SE Tyrrhenian Sea^53–55^. The two other rocks are basalt and rhyolite from Snake River Plain (USA)^56^ emplaced in intra-plate tectonic setting. The four rocks were crushed, pulverized and molten at supra liquidus temperature (1400 °C) for 4 hours in air. The obtained melts were quenched to glass and subsequently re-crushed and pulverized. This procedure was repeated twice in order to obtain homogeneous glassy materials^57^. Glasses were crushed to powders with size ranging from 500 to 200 *µ*m and then mixed together in order to obtain a series of products with intermediate compositions. For the Vulcano series, three further intermediate samples were synthesized by mixing the shoshonite and the rhyolite end-members in different weight proportions, following the procedure used for the end-member ones. Thereby, five glass compositions with variable shoshonite:rhyolite ratios of: 100:0, 70:30, 50:50, 30:70 and 0:100 (here named as S, S7, S5, S3, RS, respectively) have been prepared. Similarly, six samples were synthesized by mixing the basalt:rhyolite of the Snake River Plain compositions in the following ratios: 100:0, 80:20, 60:40, 40:60, 20:80 and 0:100 (here named B, B8, B6, B4, B2, RB). For the spectral analysis, glasses were crushed and sieved down to <25 *µ*m. This grain size limit provides good contrast in the spectral response when working in the mid-IR range^40,58^.

### Chemical analyses

Electron microprobe (EPMA) analyses were performed on all glasses. Major element concentrations of glasses were determined with a Cameca SX 50 at the CNR-IGAG laboratories in the Dipartimento di Scienze della Terra of Sapienza University in Rome. The EMPA is equipped with 5 wavelength-dispersive spectrometers and 12 crystals^59^. Iron speciation was calculated taking into consideration the experimental temperature of 1400 °C and an oxygen fugacity of air^60^.

### Spectral analyses

Spectroscopic characterization was performed at PSL (Planetary Spectroscopy Laboratory) at the DLR, Berlin, Germany. At PSL, two identical Bruker VERTEX 80 V Fourier transform infrared (FTIR) spectrometers are operating and equipped for spectral measurements. Bidirectional reflectance and transmittance measurements can be obtained in the extended wavelength ranging from 0.4 to 16 *µ*m. Emissivity can be measured under purging for sample surface temperature from 20 to 150 °C in 1.4–16 *µ*m spectral range. The second spectrometer allows measuring the bidirectional reflectance and transmittance of samples in the whole 1–100 *µ*m wavelength range. The high-temperature chamber of the Vertex 80 V allows heating (via an induction coil system) the samples to temperatures up to ~700 °C under vacuum conditions (medium vacuum – 10–100 Pa)^58^. Reflectance measurements were performed on our experimental samples with an evacuated spectrometer on samples at room temperature of 20 °C and the same samples after heating (above 500 °C). Emissivity measurements were performed in vacuum on heated powders with experimental temperatures of 150, 300, 450 and 600 °C.

## Supplementary information

Supplementary Dataset 1
