# Supplementary material for: Retrieving magma composition from TIR spectra: implications for terrestrial planets investigations
Source: Sci Rep. 2019 Oct 23;9:15200. doi: 10.1038/s41598-019-51543-9 (PMC6811632; doi:10.1038/s41598-019-51543-9)
Supplement: Supplementary file 1 — Supplementary Dataset 1 [file 41598_2019_51543_MOESM1_ESM.docx]

# Retrieving magma composition from TIR spectra: implications for terrestrial planets investigations

# Alessandro Pisello^1^,*, Francesco P. Vetere^1-5^, Matteo Bisolfati^1^, Alessandro Maturilli^2^, Daniele Morgavi^1^, Cristina Pauselli^1^, Gianluca Iezzi^3^, Michele Lustrino^4^, and Diego Perugini^1^

1Dept. of Physics and Geology, University of Perugia, piazza Universita` 1, 06123 Perugia, Italy

2Institute for Planetary Research, DLR, Rutherfordstrasse 2, 12489 Berlin-Adlershof, Germany

3Dipartimento di ingegneria e geologia, Universita` degli studi Chieti, via dei Vestini – Campus Universitario, 66100 Chieti

4Dipartimento di Scienze della Terra, Universita` Roma La Sapienza, Piazzale Aldo Moro, 5, 00185 Roma RM

^5^Institute of Mineralogy, Leibniz Universität Hannover, Callinstr. 3 D-30167 Hannover.

*[alessandro](mailto:alessandro.pisello@studenti.unipg.it)[.pisello@studenti.unipg.it](mailto:.pisello@studenti.unipg.it)

## Supplementary Tables

| **S.R.P.** | | | | | | | | | |
| --- | --- | --- | --- | --- | --- | --- | --- | --- | --- |
| R.T. | SiO_2_ wt% |  |  | SCFM |  |  | NBO/T |  |  |
|  | Calc. | EPMA | Discrepancy | Calc. | Eq. 2 | Discrepancy | Calc. | Eq. 1 | Discrepancy |
|  |  |  |  |  |  |  |  |  |  |
| B | 48.46 | 49.62 | 1.16 | 0.68 | 0.69 | 0.01 | 0.51 | 0.50 | -0.01 |
| B8 | 54.76 | 54.30 | -0.46 | 0.75 | 0.75 | 0.00 | 0.38 | 0.39 | 0.00 |
| B6 | 59.05 | 58.54 | -0.51 | 0.80 | 0.80 | 0.00 | 0.29 | 0.29 | 0.00 |
| B4 | 64.44 | 63.58 | -0.86 | 0.86 | 0.85 | -0.01 | 0.19 | 0.20 | 0.01 |
| B2 | 69.68 | 68.30 | -1.38 | 0.92 | 0.91 | -0.02 | 0.08 | 0.11 | 0.03 |
| RB | 71.40 | 73.11 | 1.71 | 0.94 | 0.96 | 0.02 | 0.05 | 0.02 | -0.03 |
|  |  |  |  |  |  |  |  |  |  |
| 500 °C | SiO_2_ wt% |  |  | SCFM |  |  | NBO/T |  |  |
|  | Calc. | EPMA | Discrepancy | Calc. | Eq. 2 | Discrepancy | Calc. | Eq. 1 | Discrepancy |
|  |  |  |  |  |  |  |  |  |  |
| B | 48.28 | 49.62 | 1.34 | 0.68 | 0.69 | 0.01 | 0.51 | 0.50 | -0.01 |
| B8 | 54.77 | 54.30 | -0.47 | 0.75 | 0.75 | 0.00 | 0.38 | 0.39 | 0.01 |
| B6 | 59.82 | 58.54 | -1.28 | 0.81 | 0.80 | -0.01 | 0.28 | 0.29 | 0.01 |
| B4 | 64.13 | 63.58 | -0.55 | 0.86 | 0.85 | 0.00 | 0.19 | 0.20 | 0.00 |
| B2 | 68.95 | 68.30 | -0.65 | 0.91 | 0.91 | -0.01 | 0.10 | 0.11 | 0.01 |
| RB | 71.91 | 73.11 | 1.20 | 0.95 | 0.96 | 0.01 | 0.04 | 0.02 | -0.02 |
|  |  |  |  |  |  |  |  |  |  |
| **Vulcano** | | | | | | | | | |
| R.T. | SiO_2_ wt% |  |  | SCFM |  |  | NBO/T |  |  |
|  | Calc. | EPMA | Discrepancy | Calc. | Eq. 2 | Discrepancy | Calc. | Eq. 1 | Discrepancy |
|  |  |  |  |  |  |  |  |  |  |
| S | 54.39 | 54.63 | 0.24 | 0.79 | 0.79 | 0.00 | 0.35 | 0.36 | 0.01 |
| S7 | 60.84 | 60.76 | -0.08 | 0.85 | 0.85 | 0.00 | 0.24 | 0.24 | -0.01 |
| S5 | 64.83 | 64.80 | -0.03 | 0.89 | 0.89 | 0.00 | 0.18 | 0.17 | -0.01 |
| S3 | 69.29 | 68.90 | -0.39 | 0.93 | 0.92 | 0.00 | 0.10 | 0.11 | 0.01 |
| RS | 74.18 | 74.57 | 0.39 | 0.97 | 0.97 | 0.00 | 0.02 | 0.02 | 0.00 |
|  |  |  |  |  |  |  |  |  |  |
| 500 °C | SiO_2_ wt% |  |  | SCFM |  |  | NBO/T |  |  |
|  | Calc. | EPMA | Discrepancy | Calc. | Eq. 2 | Discrepancy | Calc. | Eq. 1 | Discrepancy |
|  |  |  |  |  |  |  |  |  |  |
| S | 54.11 | 54.63 | 0.52 | 0.78 | 0.79 | 0.00 | 0.35 | 0.36 | 0.00 |
| S7 | 61.04 | 60.76 | -0.28 | 0.85 | 0.85 | 0.00 | 0.24 | 0.24 | 0.00 |
| S5 | 65.10 | 64.80 | -0.30 | 0.89 | 0.89 | 0.00 | 0.17 | 0.17 | 0.00 |
| S3 | 68.43 | 68.90 | 0.47 | 0.92 | 0.92 | 0.00 | 0.11 | 0.11 | -0.01 |
| RS | 74.29 | 74.57 | 0.28 | 0.98 | 0.97 | 0.00 | 0.01 | 0.02 | 0.01 |
|  |  |  |  |  |  |  |  |  |  |
|  |  |  |  |  |  |  |  |  |  |
|  |  |  |  |  |  |  |  |  |  |

Table S1: Comparison between SiO_2_ content (normalized from EPMA measurements), SCFM (Eq. 2) and NBO/T (Eq. 1) and the related values retrieved from CF through interpolation equations shown in Table 3, from reflectance spectra at Room Temperature and at 500°C, separately.

| **S.R.P** | | SiO_2_ wt% | | | SCFM | | | NBO/T | | |
| --- | --- | --- | --- | --- | --- | --- | --- | --- | --- | --- |
|  |  | Calc. | EPMA | Discrepancy | Calc. | Eq. 2 | Discrepancy | Calc. | Eq. 1 | Discrepancy |
|  |  |  |  |  |  |  |  |  |  |  |
| R.T. | B | 72.04 | 73.11 | 1.07 | 0.95 | 0.96 | 0.01 | 0.02 | 0.02 | 0.00 |
|  | B8 | 69.15 | 68.30 | -0.85 | 0.92 | 0.91 | -0.01 | 0.08 | 0.11 | 0.03 |
|  | B6 | 64.43 | 63.58 | -0.85 | 0.86 | 0.85 | -0.01 | 0.17 | 0.20 | 0.02 |
|  | B4 | 60.20 | 58.54 | -1.66 | 0.81 | 0.80 | -0.02 | 0.26 | 0.29 | 0.03 |
|  | B2 | 55.25 | 54.30 | -0.95 | 0.76 | 0.75 | -0.01 | 0.36 | 0.39 | 0.03 |
|  | RB | 48.90 | 49.62 | 0.72 | 0.68 | 0.69 | 0.00 | 0.48 | 0.50 | 0.02 |
| 500 °C | B | 70.89 | 73.11 | 2.22 | 0.94 | 0.96 | 0.02 | 0.05 | 0.02 | -0.03 |
|  | B8 | 69.15 | 68.30 | -0.85 | 0.92 | 0.91 | -0.01 | 0.08 | 0.11 | 0.03 |
|  | B6 | 63.83 | 63.58 | -0.25 | 0.86 | 0.85 | 0.00 | 0.19 | 0.20 | 0.01 |
|  | B4 | 58.36 | 58.54 | 0.18 | 0.79 | 0.80 | 0.00 | 0.29 | 0.29 | 0.00 |
|  | B2 | 54.00 | 54.30 | 0.30 | 0.74 | 0.75 | 0.00 | 0.38 | 0.39 | 0.00 |
|  | RB | 47.60 | 49.62 | 2.02 | 0.67 | 0.69 | 0.02 | 0.51 | 0.50 | -0.01 |
|  |  |  |  |  |  |  |  |  |  |  |
| **Vulcano** | | SiO_2_ wt% | | | SCFM | | | NBO/T | | |
|  |  | Calc. | EPMA | Discrepancy | Calc. | Eq. 2 | Discrepancy | Calc. | Eq. 1 | Discrepancy |
|  |  |  |  |  |  |  |  |  |  |  |
| R.T. | S | 53.29 | 54.63 | 1.34 | 0.78 | 0.79 | 0.01 | 0.37 | 0.36 | 0.01 |
|  | S7 | 60.30 | 60.76 | 0.46 | 0.84 | 0.85 | 0.01 | 0.25 | 0.24 | 0.01 |
|  | S5 | 64.64 | 64.80 | 0.16 | 0.88 | 0.89 | 0.00 | 0.18 | 0.17 | 0.01 |
|  | S3 | 69.49 | 68.90 | -0.59 | 0.93 | 0.92 | -0.01 | 0.10 | 0.11 | -0.01 |
|  | RS | 74.81 | 74.57 | -0.24 | 0.98 | 0.97 | -0.01 | 0.01 | 0.02 | -0.01 |
| 500 °C | S | 55.23 | 54.63 | -0.60 | 0.79 | 0.79 | -0.01 | 0.34 | 0.36 | -0.02 |
|  | S7 | 61.55 | 60.76 | -0.79 | 0.85 | 0.85 | -0.01 | 0.23 | 0.24 | -0.01 |
|  | S5 | 65.26 | 64.80 | -0.46 | 0.89 | 0.89 | 0.00 | 0.17 | 0.17 | 0.00 |
|  | S3 | 68.29 | 68.90 | 0.61 | 0.92 | 0.92 | 0.00 | 0.12 | 0.11 | 0.01 |
|  | RS | 73.64 | 74.57 | 0.93 | 0.97 | 0.97 | 0.01 | 0.03 | 0.02 | 0.01 |

Table S2: Comparison between SiO_2_ content (normalized from EPMA measurements), SCFM (Eq. 2) and NBO/T (Eq. 1) and the related values retrieved from CF through interpolation equations shown in Table 3 from reflectance spectra, taking into account both acquisition temperature together.

|  | |  | | **S.R.P** | |  | |  | |
| --- | --- | --- | --- | --- | --- | --- | --- | --- | --- |
|  | | **150 °C** | | **300 °C** | | **450 °C** | | **600 °C** | |
| **SiO_2_ wt%** | |  | |  | |  | |  | |
|  | EPMA | Calc. | Discrepancy | Calc. | Discrepancy | Calc. | Discrepancy | Calc. | Discrepancy |
|  |  |  |  |  |  |  |  |  |  |
| B | 49.62 | 48.90 | -0.72 | 48.29 | -1.33 | 49.22 | -0.40 | 47.14 | -2.48 |
| B8 | 54.30 | 54.49 | 0.19 | 54.83 | 0.53 | 53.74 | -0.56 | 54.14 | -0.16 |
| B6 | 58.54 | 59.37 | 0.83 | 60.10 | 1.56 | 60.33 | 1.79 | 61.46 | 2.92 |
| B4 | 63.58 | 63.09 | -0.49 | 62.68 | -0.90 | 62.47 | -1.11 | 64.20 | 0.62 |
| B2 | 68.30 | 68.26 | -0.04 | 68.22 | -0.08 | 67.71 | -0.59 | 69.03 | 0.73 |
| RB | 73.11 | 72.78 | -0.33 | 72.63 | -0.48 | 73.31 | 0.20 | 70.62 | -2.49 |
|  |  |  |  |  |  |  |  |  |  |
| **NBO/T** |  |  |  |  |  |  |  |  |  |
|  | Eq. 1 | Calc. | Discrepancy | Calc. | Discrepancy | Calc. | Discrepancy | Calc. | Discrepancy |
|  |  |  |  |  |  |  |  |  |  |
| B | 0.50 | 0.50 | 0.00 | 0.51 | 0.01 | 0.49 | -0.01 | 0.53 | 0.03 |
| B8 | 0.39 | 0.38 | 0.00 | 0.38 | -0.01 | 0.40 | 0.01 | 0.39 | 0.00 |
| B6 | 0.29 | 0.29 | -0.01 | 0.27 | -0.02 | 0.27 | -0.02 | 0.24 | -0.05 |
| B4 | 0.20 | 0.21 | 0.01 | 0.22 | 0.02 | 0.22 | 0.03 | 0.19 | -0.01 |
| B2 | 0.11 | 0.11 | 0.00 | 0.11 | 0.00 | 0.12 | 0.01 | 0.09 | -0.02 |
| RB | 0.02 | 0.01 | -0.01 | 0.02 | 0.00 | 0.00 | -0.02 | 0.06 | 0.05 |
|  |  |  |  |  |  |  |  |  |  |
| **SCFM** |  |  |  |  |  |  |  |  |  |
|  | Eq. 2 | Calc. | Discrepancy | Calc. | Discrepancy | Calc. | Discrepancy | Calc. | Discrepancy |
|  |  |  |  |  |  |  |  |  |  |
| B | 0.69 | 0.68 | 0.00 | 0.68 | -0.01 | 0.69 | 0.00 | 0.66 | -0.02 |
| B8 | 0.75 | 0.75 | 0.00 | 0.75 | 0.01 | 0.74 | -0.01 | 0.75 | 0.00 |
| B6 | 0.80 | 0.80 | 0.01 | 0.81 | 0.02 | 0.82 | 0.02 | 0.83 | 0.03 |
| B4 | 0.85 | 0.85 | -0.01 | 0.84 | -0.01 | 0.84 | -0.01 | 0.86 | 0.01 |
| B2 | 0.91 | 0.91 | 0.00 | 0.91 | 0.00 | 0.90 | -0.01 | 0.92 | 0.01 |
| RB | 0.96 | 0.96 | 0.00 | 0.96 | 0.00 | 0.97 | 0.01 | 0.93 | -0.03 |

Table S3: Comparison between SiO_2_ content (normalized from EPMA measurements), SCFM (Eq. 2) and NBO/T (Eq. 1) and the related values retrieved from CF through interpolation equations shown in Table 3, for emissivity spectra for the Snake River Plate series, separately for each acquisition temperature.

| **Vulcano** | | | | | | | | | |
| --- | --- | --- | --- | --- | --- | --- | --- | --- | --- |
|  | | **150 °C** | | **300 °C** | | **450 °C** | | **600 °C** | |
| **SiO_2_ wt%** | |  |  |  |  |  |  |  |  |
|  | EPMA | Calc. | Discrepancy | Calc. | Discrepancy | Calc. | Discrepancy | Calc. | Discrepancy |
|  |  |  |  |  |  |  |  |  |  |
| S | 54.63 | 53.39 | -1.24 | 53.93 | -0.70 | 53.79 | -0.84 | 52.15 | -2.48 |
| S7 | 60.76 | 61.47 | 0.71 | 60.52 | -0.24 | 59.73 | -1.03 | 61.98 | 1.22 |
| S5 | 64.80 | 65.38 | 0.58 | 66.32 | 1.52 | 68.32 | 3.52 | 67.31 | 2.51 |
| S3 | 68.90 | 70.30 | 1.40 | 69.16 | 0.26 | 68.88 | -0.02 | 70.21 | 1.31 |
| RS | 74.57 | 72.98 | -1.59 | 73.62 | -0.95 | 73.30 | -1.27 | 71.94 | -2.63 |
|  |  |  |  |  |  |  |  |  |  |
| **NBO/T** |  |  |  |  |  |  |  |  |  |
|  | Eq. 1 | Calc. | Discrepancy | Calc. | Discrepancy | Calc. | Discrepancy | Calc. | Discrepancy |
|  |  |  |  |  |  |  |  |  |  |
| S | 0.39 | 0.37 | -0.02 | 0.36 | -0.03 | 0.36 | -0.02 | 0.39 | 0.00 |
| S7 | 0.29 | 0.23 | -0.06 | 0.25 | -0.04 | 0.26 | -0.03 | 0.22 | -0.07 |
| S5 | 0.20 | 0.17 | -0.03 | 0.15 | -0.05 | 0.12 | -0.08 | 0.13 | -0.06 |
| S3 | 0.11 | 0.08 | -0.02 | 0.10 | 0.00 | 0.11 | 0.00 | 0.09 | -0.02 |
| RS | 0.02 | 0.04 | 0.02 | 0.03 | 0.01 | 0.04 | 0.01 | 0.06 | 0.04 |
|  |  |  |  |  |  |  |  |  |  |
| **SCFM** |  |  |  |  |  |  |  |  |  |
|  | Eq. 2 | Calc. | Discrepancy | Calc. | Discrepancy | Calc. | Discrepancy | Calc. | Discrepancy |
|  |  |  |  |  |  |  |  |  |  |
| S | 0.79 | 0.78 | -0.01 | 0.78 | 0.00 | 0.78 | 0.00 | 0.77 | -0.02 |
| S7 | 0.85 | 0.85 | 0.00 | 0.84 | -0.01 | 0.84 | -0.01 | 0.86 | 0.01 |
| S5 | 0.89 | 0.89 | 0.00 | 0.90 | 0.01 | 0.92 | 0.03 | 0.91 | 0.02 |
| S3 | 0.92 | 0.94 | 0.01 | 0.93 | 0.00 | 0.92 | 0.00 | 0.94 | 0.01 |
| RS | 0.97 | 0.96 | -0.01 | 0.97 | -0.01 | 0.96 | -0.01 | 0.95 | -0.02 |

Table S4: Comparison between SiO_2_ content (normalized from EPMA measurements), SCFM (Eq. 2) and NBO/T (Eq. 1) and the related values retrieved from CF through interpolation equations shown in Table 3, for emissivity spectra for the Vulcano series, separately for each acquisition temperature.
